# Circulating Metabolite Signatures Associated With Amyloid Positivity, Cognitive Performance and Plasma P-tau181 Levels in Cognitively Unimpaired Older Adults

**DOI:** 10.1007/s12035-026-06184-1

**Published:** 2026-09-14

**Authors:** Tahmida Sharmin, James D. Doecke, Pratishtha Chatterjee, Hamid R. Sohrabi, Henrik Zetterberg, Nicholas J. Ashton, Manohar L. Garg, Kaj Blennow, Ralph N. Martins

**Affiliations:** 1 Macquarie Medical School, Macquarie University, Macquarie Park, NSW Australia; 2 Department of Pharmacy, University of Rajshahi, Rajshahi, Bangladesh; 3 Australian eHealth Research Centre, CSIRO, Brisbane, QLD Australia; 4 School of Medical and Health Sciences, Edith Cowan University, Perth, WA Australia; 5 Florey Institute of Neuroscience and Mental Health, University of Melbourne, Melbourne, VIC Australia; 6 Department of Neuroscience, Monash University, Melbourne, VIC Australia; 7 Centre for Healthy Ageing, Health Futures Institute, Murdoch University, Murdoch, WA Australia; 8 School of Psychology, Murdoch University, Murdoch, WA Australia; 9 Alzheimer’s Research Australia, Nedlands, WA Australia; 10 Department of Psychiatry and Neurochemistry, University of Gothenburg, Mölndal, Sweden; 11 Clinical Neurochemistry Laboratory, Sahlgrenska University Hospital, Mölndal, Sweden; 12 Department of Neurodegenerative Disease, Institute of Neurology, UCL, Queen Square, London, UK; 13 UK Dementia Research Institute, UCL, London, UK; 14 Hong Kong Center for Neurodegenerative Diseases, Clear Water Bay, Hong Kong, China; 15 Wisconsin Alzheimer’s Disease Research Center, University of Wisconsin School of Medicine and Public Health, University of Wisconsin-Madison, Madison, WI USA; 16 Department of Pathology and Laboratory Medicine, University of Wisconsin School of Medicine and Public Health, University of Wisconsin-Madison, Madison, WI USA; 17 Centre for Brain Research, Indian Institute of Science, Bangalore, India; 18 Department of Old Age Psychiatry, Institute of Psychiatry, Psychology & Neuroscience, King’s College London, London, UK; 19 Lifespan Health and Wellbeing Research Centre, Macquarie University, Macquarie Park, New South Wales Australia

**Keywords:** Preclinical Alzheimer’s disease, Blood-based biomarkers, Circulating metabolites, Amyloid pathology, Cognitive performance, Plasma P-tau181

## Abstract

Emerging evidence suggests altered circulating metabolite levels in individuals with Alzheimer’s disease (AD) and/or mild cognitive impairment (MCI) compared to healthy controls; how these metabolites alter with early cortical amyloidosis—before symptom onset—remains unclear. This cross-sectional study aimed to investigate plasma metabolites obtained through targeted profiling using the Biocrates AbsoluteIDQ® p400 HR kit. The study cohort comprises 100 cognitively unimpaired (CU) older adults, stratified into CU Aβ− (negative positron emission tomography [PET]-amyloid-beta [Aβ]) and CU Aβ+ (positive PET-Aβ) groups. Significantly altered metabolites were further analysed for associations across amyloid plaque deposition, cognitive performance and plasma tau phosphorylated at threonine 181 (P-tau181) levels and for their ability to differentiate cortical PET-Aβ status in CU older adults. Significant between-group differences were observed in eighteen metabolites from phosphatidylcholine (PC), lysophosphatidylcholine (LPC) and ceramide (Cer) classes, wherein significantly lower levels of PC species and higher levels of LPC(17:1) and Cer(43:1) were detected in CU Aβ+ individuals compared to CU Aβ−. Notably, the differences became more pronounced when PC-to-LPC ratios were analysed. Across the spectrum, lower PC-to-LPC ratios were associated with greater cortical amyloid deposition, poorer cognitive performance and higher plasma P-tau181 levels. Elevated Cer(43:1) levels were also associated with higher cortical amyloid deposition. Upon receiver operating characteristic (ROC) analyses, these metabolite panels significantly distinguished CU Aβ  individuals from CU Aβ−, demonstrating good to excellent diagnostic performance. Given their association with membrane damage, synaptic dysfunction, oxidative stress, neuroinflammation and apoptosis, these metabolites may represent potential biomarkers reflecting early AD-related biochemical changes in CU older adults.

**Supplementary Information:**

The online version contains supplementary material available at https://doi.org/10.1007/s12035-026-06184-1.

## Introduction

Extracellular amyloid plaques composed of amyloid-β (Aβ) peptides and intracellular neurofibrillary tangles (NFTs) made up of hyperphosphorylated tau proteins, along with brain atrophy, represent the key neuropathological features of Alzheimer’s disease (AD) [1, 2]. The pathophysiological mechanisms contributing to this devastating disorder are multifactorial and involve several critical factors [3, 4]. Key contributors include the production and insufficient clearance of toxic Aβ, disruption of neuronal transport resulting from the accumulation of tau tangles, neuronal damage as a result of Aβ− aggregation, compromised blood circulation and oxygen delivery, excessive free radical generation, immune system activation and chronic inflammation, in conjunction with several other AD-associated changes, leading towards the gradual loss of synapses and neurons over an extended preclinical period (nearly two to three decades) [5–7], which eventually manifests through gradual deficits in cognition and memory performance [1, 6–8]. Therefore, early and precise diagnosis of AD before symptoms manifest is crucial for initiating timely preventive strategies and providing effective therapies. Consequently, developing affordable, less-invasive and easily accessible blood-based biomarkers that can track specific disease-related pathways is vital for early intervention and delayed AD progression.

Metabolomics involves the comprehensive analysis of metabolites, which are small molecules—typically less than 1.5 KDa—such as amino acids, lipids, sugars and nucleotides, that are present in cells, tissues or biological fluids, including blood, cerebrospinal fluid and urine [9, 10]. Metabolites serve as valuable indicators of both physiological and pathological conditions, as they reflect the dynamic biochemical processes occurring within the body. Their analysis can provide crucial insights for understanding various health conditions [10–13]. Previous research investigating metabolic dysregulations linked to neurodegeneration and cognitive decline has used a range of analytical methodologies [14–17]. Notably, studies focusing on AD biomarkers have utilised both targeted and untargeted metabolite profiling approaches and found alterations in the plasma metabolite levels of individuals with AD and/or mild cognitive impairment (MCI) compared to healthy individuals [18–23]. Further, Chatterjee and colleagues [24] reported altered lipid metabolism in *Presenilin1* mutation carriers compared to non-carriers in the context of autosomal dominant AD. Nevertheless, the changes in circulating metabolite levels with the early cortical amyloidosis during the asymptomatic phase in sporadic AD have yet to be thoroughly investigated.

The current research aimed at investigating the cross-sectional alterations in circulating metabolite levels associated with early cortical Aβ aggregation ahead of symptom onset using a targeted metabolomic analysis platform and a cognitively unimpaired (CU) older adult cohort; therefore, the current investigation compared the levels of the circulating metabolites between cognitively unimpaired individuals with negative PET-Aβ (CU Aβ−) and those with positive PET-Aβ (CU Aβ+). Accordingly, this study employed the AbsoluteIDQ® p400 HR kit (Biocrates life sciences ag) on a mass spectrometry–based platform—targeting specific metabolite classes, including amino acid, biogenic amine, hexose, phosphatidylcholine, lysophosphatidylcholine, sphingomyelin, ceramide, acylcarnitine, diglyceride, triglyceride and cholesteryl esters—connected with specific pathophysiological processes including insulin resistance, oxidative stress, inflammation, neurotransmitter homeostasis, mitochondrial dysfunction, neurodegeneration, lipid disturbance and aberrant signal transduction [25–27]. In addition, the identified circulating metabolites were examined for differential associations across the spectrum of amyloid plaque deposition, cognitive performance and plasma P-tau181 levels. Furthermore, the circulating metabolite signatures were evaluated for their classification performance in discriminating cortical PET-Aβ status.

## Methods

### Study Participants

The participants were drawn from the Kerr Anglican Retirement Village Initiative in Ageing Health (KARVIAH) cohort, comprising residents of Anglicare in New South Wales (NSW), Australia. Of the 200 initial volunteers, 134 individuals (*n* = 134) met the inclusion and exclusion criteria, as outlined previously [27–29]. In summary, participants in the KARVIAH cohort were community-dwelling older adults aged 65–90 years who were fluent in English and had adequate or corrected vision and hearing. All participants were in good general health, had no known significant cerebrovascular disease and demonstrated no dementia or objective cognitive impairment, defined by a Montreal Cognitive Assessment (MoCA) score ≥ 26. The assessment (range = 0–30 points) involved evaluating various cognitive domains, including short-term memory, learning trials, delayed recall, visuospatial skills, three-dimensional cube reproduction, trail-making, verbal fluency, abstraction and attention [28]. For participants with MoCA scores ranging from 18 to 25, the study neuropsychologist conducted individualised evaluations, adjusting for age and education norms based on the guidelines of Rossetti and colleagues [30]. Individuals were excluded if they had a prior diagnosis of dementia according to the National Institute on Aging–Alzheimer’s Association criteria, a history of schizophrenia or bipolar disorder, stroke, severe or extremely severe depression based on the Depression Anxiety and Stress Scale (DASS) or uncontrolled hypertension (systolic blood pressure > 170 mmHg or diastolic blood pressure > 100 mmHg). Neuropsychological evaluations, neuroimaging and blood sample collection were eventually conducted for 105 participants, among whom 100 were classified as cognitively unimpaired based on a Mini-Mental State Examination (MMSE) score of ≥ 26 [31] and were included in the study. All participants provided informed consent ahead of participation, and the study received ethical approval from the Bellberry Human Research Ethics Committee, Australia (approval reference no. 2012-09-1086) and the Macquarie University Human Research Ethics Committee (approval reference no. 520231431450224). This research was not pre-registered, and the entire eligible cohort meeting the screening criteria was utilised.

### Assessments of Cortical PET-Aβ and Hippocampal Volume

All participants underwent positron emission tomography (PET) using 18F-florbetaben (FBB) to measure cortical Aβ load [32–34] and magnetic resonance imaging (MRI) for hippocampal volume [35, 36] within 3 months of blood sample collection at Macquarie Medical Imaging, NSW, Australia. The FBB-PET procedure involved a 20-min static scan (4 × 5-min dynamic frames) starting 50 min after intravenous FBB injection. According to the methods outlined by Bourgeat et al. [37] and Zhou et al. [38], the mean standard uptake value ratio (SUVR) across several neocortical regions, including (a) the frontal, (b) superior parietal, (c) lateral temporal, (d) lateral occipital and (e) anterior and posterior cingulate, was calculated using CapAIBL image processing software to estimate global cortical Aβ load, with the cerebellar cortex used as the reference region. As previously reported [27–29, 32], participants were classified according to their cortical PET-Aβ load using an SUVR threshold of 1.35. CU individuals with an SUVR below 1.35 were categorised as having a negative PET-Aβ (CU Aβ−), while those with an SUVR of 1.35 or higher were classified as having a positive load of PET-Aβ (CU Aβ+). In addition, participants underwent MRI scanning using a General Electric 3-Tesla scanner (Model 750 W), as described by Goozee et al. [32]. The acquired T1-weighted MRI images were first normalised for total intracranial volume, which includes grey matter, white matter and cerebrospinal fluid, before being used to calculate hippocampal volumes.

### Neuropsychological Evaluations

The study participants completed an extensive series of neuropsychological assessments [39]—namely the MoCA [30], the MMSE [31], the Memory Complaint Questionnaire (MAC-Q) [40], the Rey Auditory Verbal Learning Test (RAVLT) List A, RAVLT short delay, and RAVLT long delay [41], the Logical Memory I & II (WMS-III; Story A only) [42], the Rey Complex Figure Test (RCFT) 3-minute and 30-minute delays [43], the WAIS-III Digit Span backward [44], the WAIS-III Digit Symbol Substitution Test (DSST) [45], the D-KEFS tasks: Category Fluency (Boys' Names) and Category Switching (Fruits and Furniture) [46], the Controlled Oral Word Association Test [47], the Stroop Test (Victoria version) [48], the Boston Naming Test [49], the Wechsler Test of Adult Reading [50] and the Depression, Anxiety, and Stress Scales (DASS) [51] were evaluated. Composite scores for cognitive measures, including verbal and visual episodic memory, as well as working memory and executive function, were calculated and then combined with MMSE scores to derive the global composite scores. Each cognitive composite measure was calculated using the mean *z*-scores of relevant neuropsychological tests, as detailed in Sharmin et al. [27]. To examine the impact of metabolite alterations on cognitive performance, participants were stratified using tertiles of the global composite *z*-scores [52, 53]. The lowest tertile represented poor cognitive performance, while the highest tertile indicated superior cognitive function.

### Blood Collection, *APOE* Genotyping, Plasma P-tau181 Quantification and Metabolomic Measurements

Blood samples were collected via standard serologic procedures from all participants in the morning after a minimum of 10 h of overnight fasting. The collected samples were then processed and stored at −80 °C, as per the protocol described by Goozee et al. [32]. *APOE* genotyping was conducted using purified genomic DNA extracted from 0.5 ml of whole blood. As Goozee et al. [32] outlined, TaqMan SNP genotyping assays were used to detect the three *APOE* variants: ε2, ε3 and ε4. However, adhering to the manufacturer’s protocol, duplicate testing of 5% of samples confirmed 100% inter- and intra-assay consistency. Plasma P-tau181 levels were quantified using an in-house single-molecule array (Simoa)–based assay developed at the University of Gothenburg, Sweden [27, 29]. Calibrators, controls and samples were analysed in duplicate. The lower limit of quantification was 1 pg/ml, with an average coefficient of variation (CV) of 8%. In this cross-sectional analysis, 97 samples were available, including 65 CU Aβ− and 32 CU Aβ+ participants. As mentioned earlier by Chatterjee et al. [25], targeted profiling of the endogenous plasma metabolites was performed in 2019 using the Biocrates AbsoluteIDQ® p400 HR kit (Innsbruck, Austria). The assay platform employed ultra-high-performance liquid chromatography-mass spectrometry (UHPLC-MS) by integrating the Thermo Scientific™ Vanquish™ UHPLC system with the Thermo Scientific™ Q Exactive Plus™ HRAM-MS. Following the manufacturer’s protocol, all samples were deidentified, randomised and blinded before metabolomic profiling. Although the AbsoluteIDQ® p400 HR kit can target up to 408 metabolite species, those with more than 40% of measurements falling below the lower limit of detection (LLOD) were excluded [54]. As a result, 216 metabolites were retained for further analysis, aligning with the approach used by Thompson et al. [55]. Test samples were analysed in singlicate runs, with five manufacturer-provided quality control 2 (QC2) samples placed on each plate (every 19–20 test samples) to ensure consistent quantitative performance across plates and to normalise intraplate and interplate variations [25, 27].

### Statistical Analyses

Study participant characteristics were presented as counts and percentages for categorical features and as mean ± standard deviation (s.d.) for continuous features. The demographic, neuroimaging and cognitive parameters were compared between the CU Aβ− and CU Aβ+ groups employing independent-samples *t*-tests or chi-square tests, as appropriate. While assessing normality, the dependent variables were evaluated using the Kolmogorov-Smirnov and Shapiro-Wilk tests, and where necessary, subsequent logarithmic or square-root transformations were employed to approximate a normal distribution. The entire cohort met the KARVIAH study criteria and had been used in similar research [25, 27, 53]. As the entire cohort was included and the study design focused on a screening approach to identify appropriate biochemical markers for AD, no sample size calculation or outlier analysis was performed. During the initial screening of metabolites, between-group differences were assessed using independent-samples *t*-tests, as metabolite concentrations satisfied normality assumptions after data transformation. As this screening step was exploratory and hypothesis-generating, false discovery rate correction was not applied. Based on known biochemical relationships and pathway relevance, plasma phosphatidylcholine-to-lysophosphatidylcholine (PC-to-LPC) ratios were subsequently computed and examined. Between-group differences in these ratios were analysed using the Mann-Whitney *U*-tests because the ratio variables deviated from a normal distribution despite transformation; additionally, the statistical significances were further examined using the generalised linear models to account for AD-related confounding variables, including *APOE* ε4 status, sex and age, followed by false discovery rate corrections for multiple comparisons. Generalised linear models were used for an adjusted confirmatory analysis to compare disease stages while accounting for non-normally distributed outcomes. Cross-sectional relationships were deemed significant if *p*-values were < 0.05 after statistical testing. All identified circulating metabolites were further analysed for their cross-sectional relationships across the spectrum of amyloid plaque deposition (via FBB-PET tertiles) [56], cognitive performance (via global composite tertiles) [53] and plasma P-tau181 levels (via P-tau181 tertiles). Given the exploratory nature of the study and modest sample size, tertile stratification was employed to facilitate comparisons across varying levels of these AD-related measures. These analyses were conducted using Kruskal-Wallis tests or ordinary one-way ANOVA, as appropriate, followed by pairwise multiple comparisons, with false discovery rate adjustments applied using Benjamini-Hochberg corrections. Logistic regression model-fitted values were used to develop receiver operating characteristic (ROC) curves and to calculate the area under the curve (AUC). Additionally, AUC with its 95% confidence interval (CI), *p*-value, sensitivity, specificity, threshold, positive predictive value (PPV), negative predictive value (NPV) and accuracy were calculated for each of the final models. DeLong’s test was applied to compare the predictive accuracies of different AD blood biomarkers. Statistical assessments were performed using IBM SPSS (v28), GraphPad Prism (v10.4.1) and the R statistical environment (v4.1.0), with graphs generated using GraphPad Prism or R for visualisation.

## Results

### Study Participants

The demographic, neuroimaging and cognitive performance measures of the study participants are presented in Table 1. While looking at demographic characteristics, i.e. *APOE* ε4 status, sex, age, body mass index (BMI) and education status, only *APOE* ε4 status differed significantly between the CU Aβ− and CU Aβ+ individuals, as expected. No between-group difference was observed for hippocampal volume as measured from the MRI data. Additionally, there was no significant difference between the study groups in cognitive performance, as measured by MMSE and the global composite scores. Although not statistically significant (*p*-value = 0.08), global cognitive composite scores were approximately twofold lower in the CU Aβ+ group than in the CU Aβ− group, suggesting subtle cognitive differences associated with early amyloid pathology. Furthermore, AD-related plasma measure P-tau181 showed significant differences between CU Aβ− and CU Aβ+ individuals.

**Table 1:** Demographic, brain imaging, cognitive performance and plasma measures of the study participants

	Total participants (*N*)	Groups by cortical PET-Aβ buildup		*p*-value
		CU Aβ−	CU Aβ+	
**Demographic factors**				
*APOE* ε4+ status (*n* (%))	100 (65, 35)	5 (7.692)	16 (45.714)	**< 0.0001**
Female (*n* (%))	100 (65, 35)	46 (70.769)	22 (62.857)	0.419
Age (years)	100 (65, 35)	77.615 ± 5.556	79.228 ± 5.380	0.165
BMI (kg/m^2^)	100 (65, 35)	27.385 ± 4.477	28.054 ± 4.735	0.486
Education (years)	100 (65, 35)	14.846 ± 3.373	13.643 ± 2.919	0.078
**Neuroimaging factors**				
Neocortical FBB-PET SUVR	100 (65, 35)	1.158 ± 0.086	1.714 ± 0.261	**< 0.0001**
Hippocampal volume (left, %)	96 (64, 32)	0.195 ± 0.020	0.194 ± 0.019	0.805
Hippocampal volume (right, %)	96 (64, 32)	0.199 ± 0.021	0.199 ± 0.018	0.891
**Cognitive measures**				
MMSE	100 (65, 35)	28.508 ± 1.161	28.800 ± 1.106	0.225
Global composite (*z*-score)	100 (65, 35)	0.070 ± 0.488	−0.130 ± 0.625	0.080
**Plasma measure**				
Plasma P-tau181 (pg/ml)	97 (65, 32)	13.549 ± 5.693	17.273 ± 5.578	**0.001** ^$^

Table describing study participant characteristics, including categorical factors as percentages with count and continuous factors as mean ± s.d., and demonstrating values up to 3 decimal places, except for the < 0.0001 values. For between-group differences, *p*-values were calculated using the independent sample *t*-tests or chi-square tests and were considered statistically significant at *p*-values < 0.05 (in bold font). The cortical PET-Aβ was stratified using the FBB-PET SUVR cut-off 1.35 (CU Aβ−; SUVR < 1.35 and CU Aβ+; SUVR ≥ 1.35). *n* represents the count. *APOE* apolipoprotein E gene, *BMI* body mass index, *FBB *^18^F-Florbetaben, *PET* positron emission tomography, *SUVR* standardised uptake value ratio, *CU* cognitively unimpaired, *MoCA* Montreal cognitive assessment, *MMSE* mini-mental state examination. The normal distribution requirement was obtained using logarithmic transformation (^$^)

### Initial Screening for Circulating Metabolites to Compare Between CU Aβ− and CU Aβ+ Individuals

**Fig. 1:**
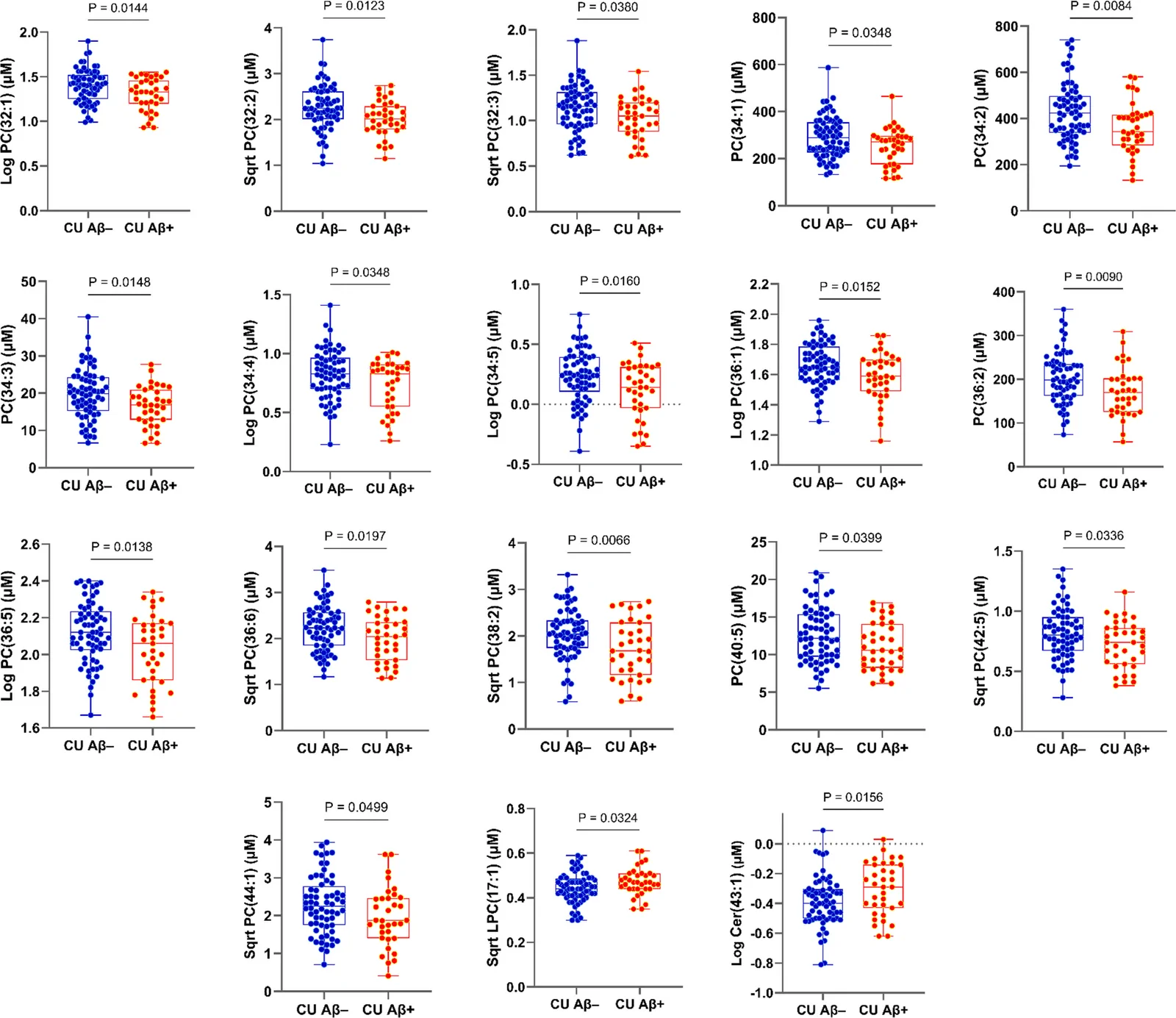
Circulating metabolites that differ significantly between CU Aβ‒ and CU Aβ+ individuals. The p-values were calculated using independent-samples t-tests. P represents p-values, whereas values <0.05 were considered statistically significant. For all metabolites, n = 65 in CU Aβ‒ and n = 35 in CU Aβ+, except for PC(44:1), where n = 64 in CU Aβ‒ and n = 34 in CU Aβ+. Each of these box and whisker plots comprises i) a box consisting of a horizontal bar representing the median and Q3 to Q1 representing the interquartile range, and ii) the whiskers, upper and lower, representing the maximum and the minimum values

Initial screening analyses of individual metabolites were performed following log or square-root transformation to approximate normality. Where distributional assumptions were met, group comparisons using independent-samples *t*-tests identified several metabolites that differed significantly between CU Aβ− and CU Aβ+ individuals (Table 2 and Fig. 1). As this step was exploratory and hypothesis-generating, false discovery rate correction was not applied. A total of eighteen circulating metabolites from the phosphatidylcholine (PC), lysophosphatidylcholine (LPC) and ceramide (Cer) classes showed significant differences between the two groups. Notably, sixteen phosphatidylcholine species, including PC(32:1), PC(32:2), PC(32:3), PC(34:1), PC(34:2), PC(34:3), PC(34:4), PC(34:5), PC(36:1), PC(36:2), PC(36:5), PC(36:6), PC(38:2), PC(40:5), PC(42:5) and PC(44:1), were significantly reduced in CU Aβ+ individuals relative to CU Aβ− individuals. Conversely, CU Aβ+ individuals exhibited significantly elevated plasma levels of LPC(17:1) and Cer(43:1) compared to controls.

**Table 2:** Circulating metabolites showing significant differences between CU Aβ− and CU Aβ+ individuals

Plasma metabolites (μM)	Total participants (*N*)	Groups by cortical PET-Aβ buildup		*p*-value
		CU Aβ−	CU Aβ+	
PC(32:1) ^$^	100 (65, 35)	27.455 ± 12.416	21.763 ± 8.254	**0.0144**
PC(32:2) ^Ϯ^	100 (65, 35)	5.365 ± 2.272	4.237 ± 1.527	**0.0123**
PC(32:3) ^Ϯ^	100 (65, 35)	1.358 ± 0.583	1.111 ± 0.460	**0.0380**
PC(34:1)	100 (65, 35)	290.385 ± 88.417	252.343 ± 77.482	**0.0348**
PC(34:2)	100 (65, 35)	428.369 ± 126.630	359.800 ± 111.439	**0.0084**
PC(34:3)	100 (65, 35)	19.845 ± 6.810	16.555 ± 5.287	**0.0148**
PC(34:4) ^$^	100 (65, 35)	7.473 ± 3.907	5.932 ± 2.483	**0.0348**
PC(34:5) ^$^	100 (65, 35)	1.926 ± 0.969	1.489 ± 0.710	**0.0160**
PC(36:1) ^$^	100 (65, 35)	48.671 ± 15.659	41.263 ± 14.206	**0.0152**
PC(36:2)	100 (65, 35)	203.440 ± 59.118	170.883 ± 56.643	**0.0090**
PC(36:5) ^$^	100 (65, 35)	140.966 ± 52.350	116.206 ± 48.100	**0.0138**
PC(36:6)^Ϯ^	100 (65, 35)	5.214 ± 2.248	4.171 ± 1.890	**0.0197**
PC(38:2) ^Ϯ^	100 (65, 35)	4.490 ± 2.235	3.293 ± 2.172	**0.0066**
PC(40:5)	100 (65, 35)	12.585 ± 3.620	11.071 ± 3.157	**0.0399**
PC(42:5) ^Ϯ^	100 (65, 35)	0.708 ± 0.362	0.554 ± 0.282	**0.0336**
PC(44:1) ^Ϯ^	98 (64, 34)	5.810 ± 3.709	4.447 ± 3.268	**0.0499**
LPC(17:1)^Ϯ^	100 (65, 35)	0.201 ± 0.058	0.227 ± 0.059	**0.0324**
Cer(43:1) ^$^	100 (65, 35)	0.440 ± 0.186	0.538 ± 0.210	**0.0156**

Plasma metabolites are presented as mean ± s.d., and values are shown up to 3 decimal places, except for *p*-values. The *p*-values were calculated using the independent sample *t*-tests, whereas *p*-values < 0.05 were considered statistically significant. As this represents an exploratory screening analysis, correction for multiple comparisons (e.g. false discovery rate) was not applied. The normal distribution requirement was obtained using square root (^Ϯ^) and logarithmic (^$^) transformation as appropriate. *PET* positron emission tomography, *PC* phosphatidylcholine, *LPC* lysophosphatidylcholine, *Cer* ceramide

### Investigation of Plasma Phosphatidylcholine-to-Lysophosphatidylcholine (PC-to-LPC) Ratios for Comparison Between CU Aβ− and CU Aβ+ Individuals

Based on known biochemical relationships and pathway relevance, plasma PC-to-LPC ratios were computed and examined, further enhancing the distinction between CU Aβ− and CU Aβ+ individuals (Table 3). As these ratios remained non-normally distributed even after transformation, nonparametric Mann-Whitney *U*-tests were used for unadjusted comparisons.

**Table 3:** Plasma PC-to-LPC ratios to compare between CU Aβ− and CU Aβ+ individuals

PC-to-LPC ratios	Groups by cortical PET-Aβ buildup		*p*-value	*p*^a^	*p*^b^
	CU Aβ− (*n* = 65)	CU Aβ+ (*n* = 35)			
PC(32:1)/LPC(17:1)	146.474 ± 82.045	97.573 ± 34.947	**0.0004**	**0.0004**	**0.0009**
PC(32:2)/LPC(17:1)	28.400 ± 13.688	19.322 ± 7.125	**0.0008**	**0.0159**	**0.0319**
PC(32:3)/LPC(17:1)	7.073 ± 3.112	4.943 ± 1.689	**0.0006**	**0.0012**	**0.0025**
PC(34:1)/LPC(17:1)	1533.606 ± 557.260	1158.893 ± 431	**0.0002**	**0.0107**	**0.0215**
PC(34:2)/LPC(17:1)	2272.045 ± 854.978	1661.581 ± 611.248	**0.0002**	**0.0232**	**0.0464**
PC(34:3)/LPC(17:1)	104.065 ± 41.839	75.047 ± 24.652	**0.0001**	**0.0029**	**0.0059**
PC(34:4)/LPC(17:1)	38.935 ± 22.720	26.303 ± 9.946	**0.0010**	**0.0003**	**0.0006**
PC(34:5)/LPC(17:1)	10.038 ± 5.204	6.684 ± 3.124	**0.0006**	**0.0106**	**0.0211**
PC(36:1)/LPC(17:1)	254.893 ± 89.026	188.575 ± 72.210	**0.0002**	**0.0033**	**0.0068**
PC(36:2)/LPC(17:1)	1082.197 ± 427.594	789.088 ± 318.861	**0.0001**	**0.0357**	0.0715
PC(36:5)/LPC(17:1)	740.338 ± 316.132	529.103 ± 230.079	**0.0003**	**0.0155**	**0.0311**
PC(36:6)/LPC(17:1)	26.994 ± 12.250	18.637 ± 8.151	**0.0005**	**0.0037**	**0.0075**
PC(38:2)/LPC(17:1)	23.568 ± 11.855	14.880 ± 9.615	**0.0003**	**0.0052**	**0.0105**
PC(40:5)/LPC(17:1)	66.542 ± 24.365	50.305 ± 14.818	**0.0004**	**0.0104**	**0.0209**
PC(42:5)/LPC(17:1)	3.724 ± 2.045	2.518 ± 1.285	**0.0020**	**0.0098**	**0.0196**
PC(44:1)/LPC(17:1)	30.339 ± 21.689	20.219 ± 15.161	**0.0086**	**0.0400**	0.0800

PC-to-LPC ratios (mean ± s.d.) to compare between CU Aβ− and CU Aβ+ individuals. All values are displayed up to 3 decimal places, except for *p*-values. The *p*-values were calculated using the nonparametric Mann-Whitney *U*-tests, and values < 0.05 were considered statistically significant; this table includes only statistically significant associations. For PC(44:1)/LPC(17:1), *n* = 64 in CU Aβ− and *n* = 34 in CU Aβ+. ‘p^a^’ represents the *p*-values calculated using the generalised linear model for correcting for confounding variables, including *APOE* ε4 status, sex and age, and ‘p^b^’ represents the *p*-values after adjusting for false discovery rate corrections for multiple comparisons. *n* represents the number of participants. *PET* positron emission tomography, *PC* phosphatidylcholine, *LPC* lysophosphatidylcholine

For adjusted confirmatory modelling, generalised linear models controlling for relevant covariates such as *APOE* ε4 status, sex and age were applied. As presented in Table 3, these ratios remained significantly associated with group status (adjusted *p*^a^ < 0.05), and following false discovery rate corrections (adjusted *p*^b^ < 0.05), retained statistical significance, indicating robust associations independent of confounding effects.

### Comparison of All Identified Circulating Metabolites Across the Spectrum of Amyloid Plaque Deposition

**Fig. 2:**
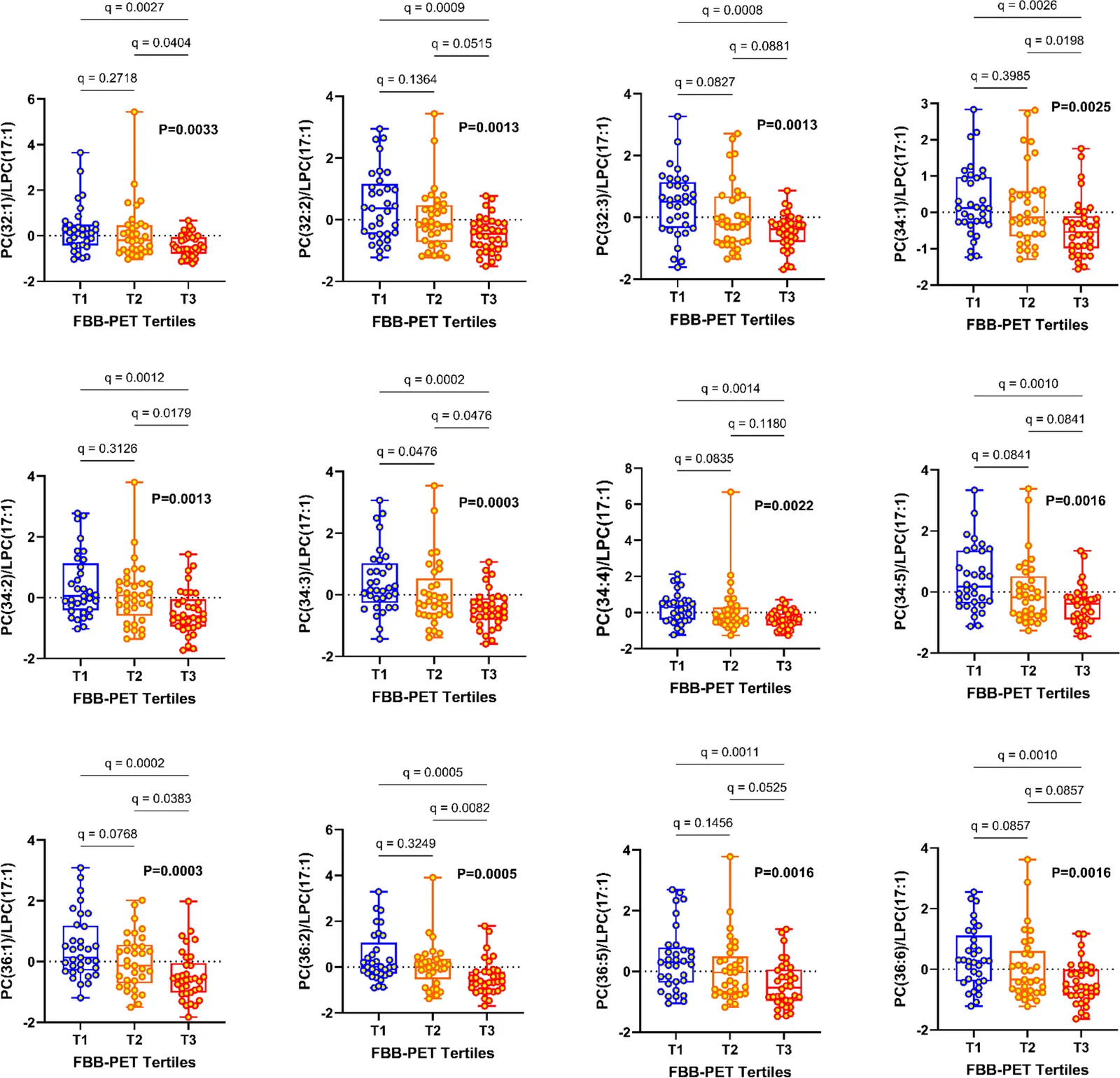

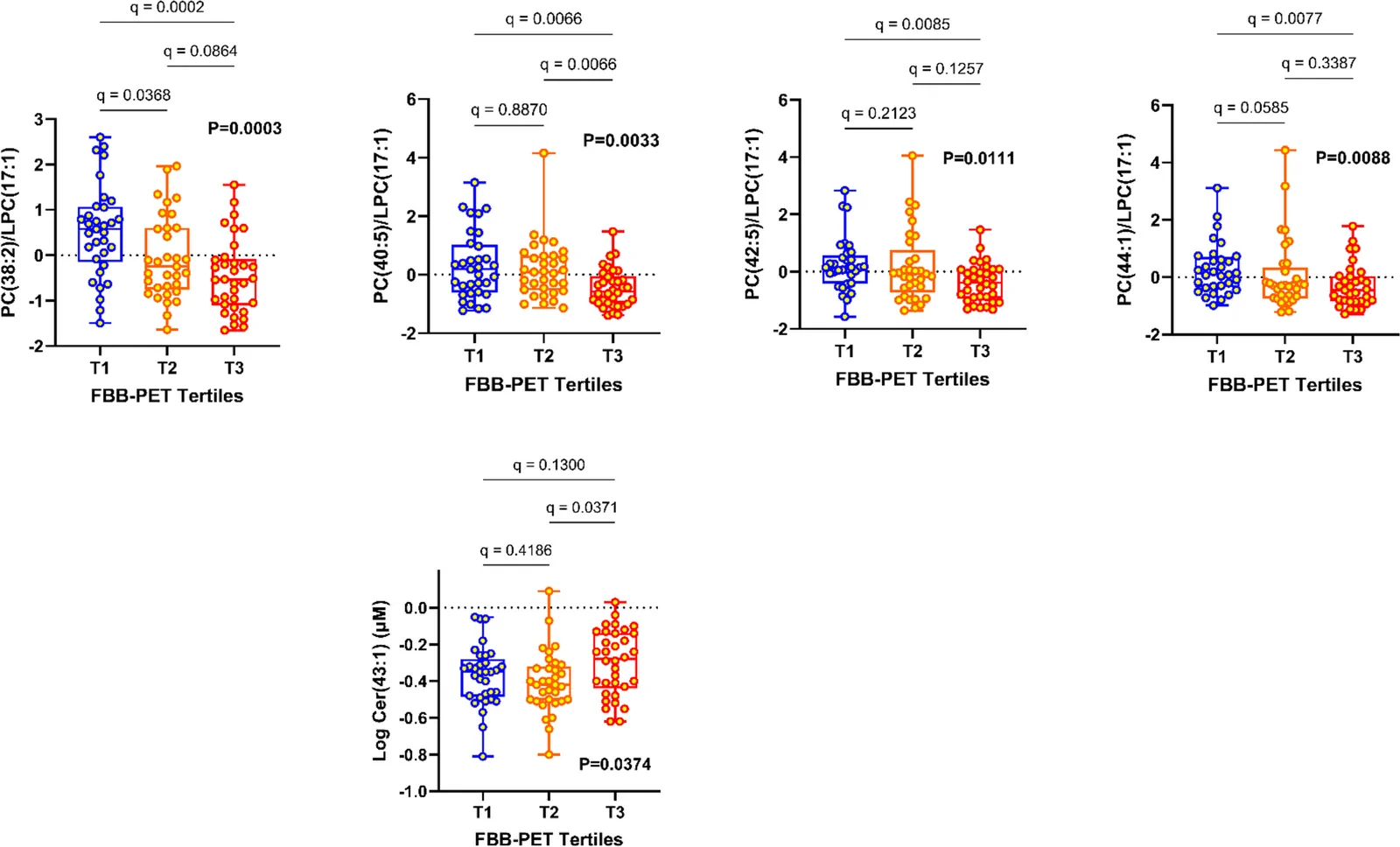
Comparison of all identified circulating metabolites across the spectrum of cortical amyloid plaque deposition. The Kruskal-Wallis test or ordinary one-way ANOVA, as appropriate, and subsequent pair-wise comparisons adjusting for a false discovery rate using Benjamini–Hochberg correction were employed to compare the metabolite levels by the FBB-PET tertiles, wherein T1 represents lower amyloid buildup (n=33, mean ± s.d= 1.088 ± 0.044), T2 represents average amyloid buildup (n=33, mean ± s.d= 1.234 ± 0.056) & T3 represents higher amyloid buildup (n=34, mean ± s.d= 1.724 ± 0.257). P represents the Kruskal-Wallis test or ordinary one-way ANOVA p-values, q value represents the "false discovery rate adjusted" p-value using Benjamini–Hochberg correction, and all p-values below 0.05 were considered statistically significant. Each of these box and whisker plots comprises i) a box consisting of a horizontal bar representing the median and Q3 to Q1 representing the interquartile range, and ii) the whiskers, upper and lower, representing the maximum and the minimum values

To explore the cross-sectional relationships between alterations in all identified metabolites—including Cer(43:1) from the initial screening and PC-to-LPC ratios from the confirmatory investigation—and cortical Aβ aggregation, metabolite levels were compared across FBB-PET tertiles. Consistent with the approach reported by Barthélemy et al. [56], tertile stratification was used to assess associations across increasing levels of amyloid accumulation. The analysis was conducted using either the nonparametric Kruskal-Wallis test or ordinary one-way ANOVA, as appropriate, followed by pairwise comparisons adjusted for false discovery rate using the Benjamini-Hochberg correction (Fig. 2). Further confirming significantly lower plasma levels of all sixteen PC-to-LPC ratios and elevated Cer(43:1) levels with higher cortical Aβ aggregation.

### Comparison of All Identified Circulating Metabolites Across the Spectrum of Cognitive Performance

**Fig. 3:**
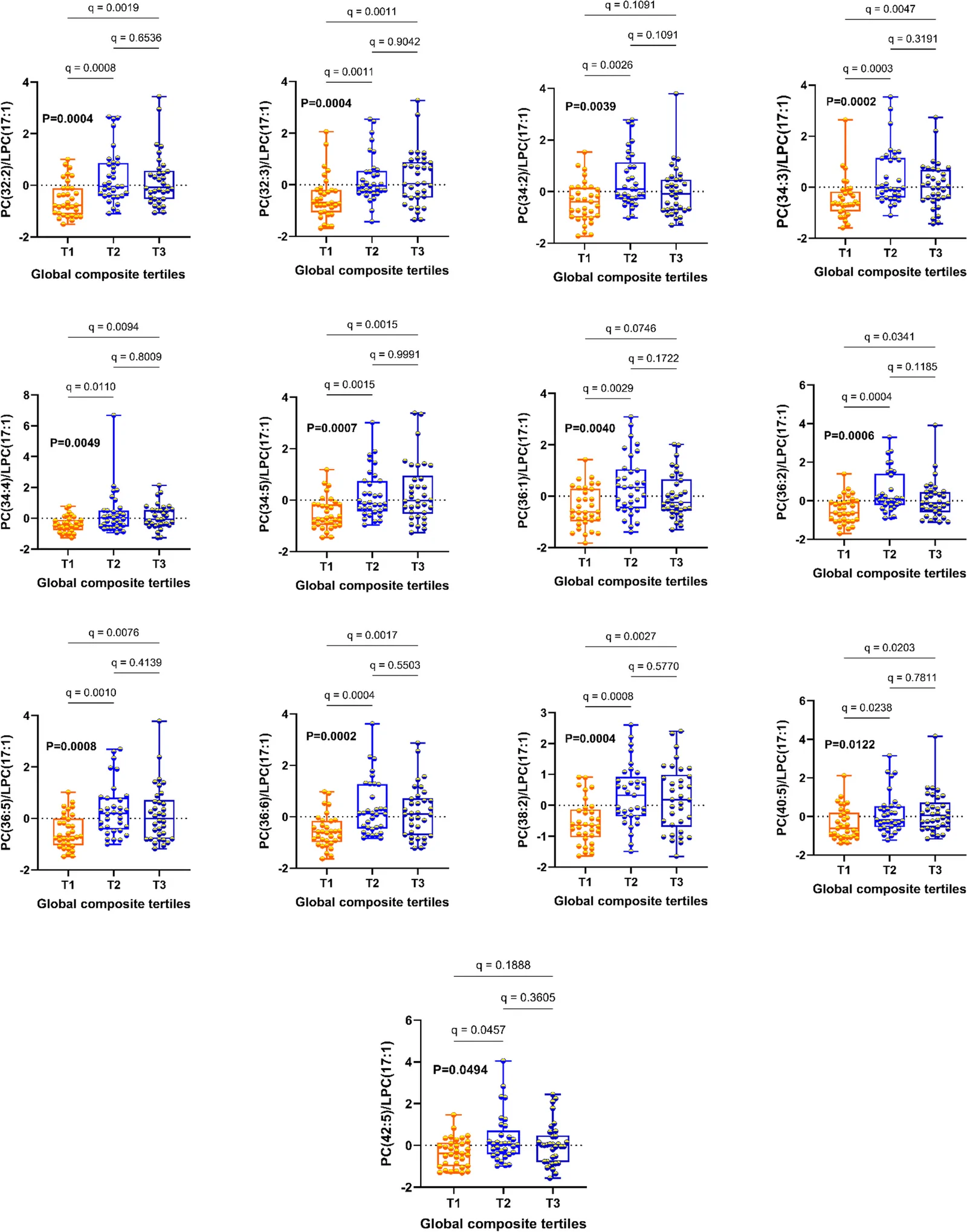
Comparison of all identified circulating metabolites across the spectrum of cognitive performance. The Kruskal-Wallis test and subsequent pair-wise comparisons adjusting for a false discovery rate using Benjamini–Hochberg correction were employed to compare the metabolite levels across the global composite tertiles, wherein T1 represents poor cognitive performance (n=33, mean ± s.d= -0.634 ± 0.282), T2 represents average cognitive performance (n=33, mean ± s.d= 0.035 ± 0.119) & T3 represents superior cognitive performance (n=34, mean ± s.d= 0.581 ± 0.224). P represents the Kruskal-Wallis test p-values, q value represents the "false discovery rate adjusted" p-value using Benjamini–Hochberg correction, and all p-values below 0.05 were considered statistically significant. Each of these box and whisker plots comprises i) a box consisting of a horizontal bar representing the median and Q3 to Q1 representing the interquartile range, and ii) the whiskers, upper and lower, representing the maximum and the minimum values

All identified circulating metabolites were analysed across tertiles of global cognitive performance, consistent with the approach previously reported in Sharmin et al. [53]. The cross-sectional relationships between metabolite signatures and cognitive performance were investigated using the Kruskal-Wallis test, followed by pairwise multiple comparisons adjusted for false discovery rate using the Benjamini-Hochberg correction (Fig. 3). Notably, thirteen PC-to-LPC ratios, including PC(32:2)/LPC(17:1), PC(32:3)/LPC(17:1), PC(34:2)/LPC(17:1), PC(34:3)/LPC(17:1), PC(34:4)/LPC(17:1), PC(34:5)/LPC(17:1), PC(36:1)/LPC(17:1), PC(36:2)/LPC(17:1), PC(36:5)/LPC(17:1), PC(36:6)/LPC(17:1), PC(38:2)/LPC(17:1), PC(40:5)/LPC(17:1) and PC(42:5)/LPC(17:1), exhibited significantly lower plasma levels accompanying poor cognitive performance.

### Associations of Identified Circulating Metabolites with Plasma P-tau181 Elevation and AD-Related Risk Factors

**Fig. 4:**
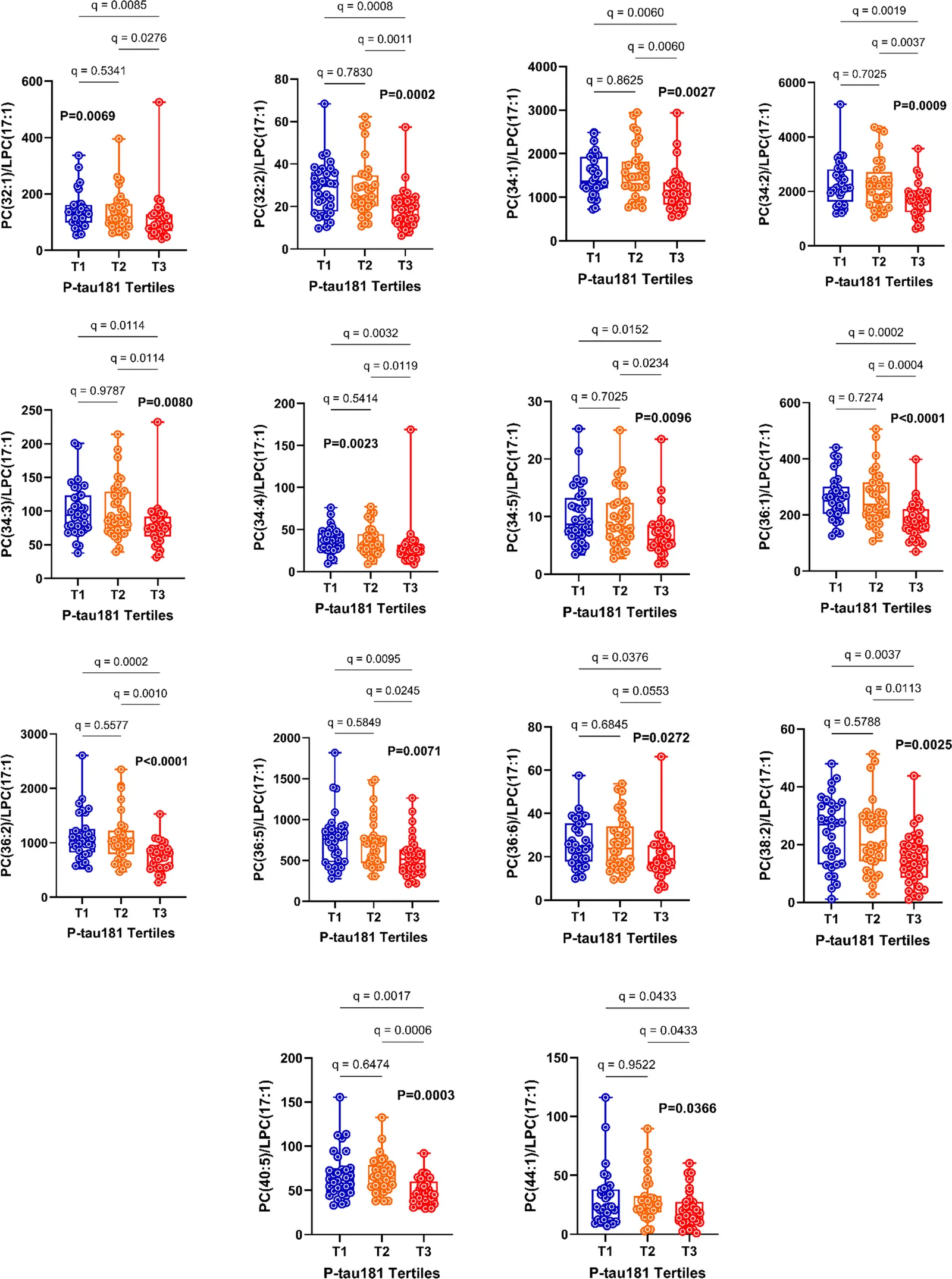
Comparison of all identified circulating metabolites across the spectrum of plasma P-tau181 elevation. The Kruskal-Wallis test and subsequent pair-wise comparisons adjusting for a false discovery rate using Benjamini–Hochberg correction were employed to compare the metabolite levels across the P-tau181 tertiles, wherein T1 represents lower plasma P-tau181 level (n=32, mean ± s.d= 9.108 ± 1.468), T2 represents average plasma P-tau181 level (n=32, mean ± s.d= 13.424 ± 1.340) & T3 represents higher plasma P-tau181 level (n=33, mean ± s.d= 21.588 ± 4.318). P represents the Kruskal-Wallis test p-values, q value represents the "false discovery rate adjusted" p-value using Benjamini–Hochberg correction, and all p-values below 0.05 were considered statistically significant. Each of these box and whisker plots comprises i) a box consisting of a horizontal bar representing the median and Q3 to Q1 representing the interquartile range, and ii) the whiskers, upper and lower, representing the maximum and the minimum values

To assess whether the identified metabolite alterations were associated with AD-related pathology beyond PET-Aβ status, all identified circulating metabolites were also analysed across the spectrum of plasma P-tau181 levels, measured by plasma P-tau181 tertiles, given that P-tau181 is an established biomarker of early amyloid-related pathology that precedes tau tangle development [27]. The cross-sectional relationships between metabolite signatures and P-tau181 elevation were investigated using the Kruskal-Wallis test, followed by pairwise comparisons adjusted for false discovery rate using the Benjamini-Hochberg correction (Fig. 4). Notably, fourteen PC-to-LPC ratios, including PC(32:1)/LPC(17:1), PC(32:2)/LPC(17:1), PC(34:1)/LPC(17:1), PC(34:2)/LPC(17:1), PC(34:3)/LPC(17:1), PC(34:4)/LPC(17:1), PC(34:5)/LPC(17:1), PC(36:1)/LPC(17:1), PC(36:2)/LPC(17:1), PC(36:5)/LPC(17:1), PC(36:6)/LPC(17:1), PC(38:2)/LPC(17:1), PC(40:5)/LPC(17:1) and PC(44:1)/LPC(17:1), exhibited significantly lower plasma levels in individuals with elevated plasma P-tau181 levels.

All identified circulating metabolites were investigated for associations with AD-related risk factors. When considering the *APOE* ε4 status, significant between-group differences were observed for PC(34:1)/LPC(17:1), PC(34:2)/LPC(17:1), PC(36:2)/LPC(17:1) and PC(42:5)/LPC(17:1) (Supplementary Table 1). When considering sex, significantly higher levels were observed in female participants for most of the PC-to-LPC ratios (Supplementary Table 2). Significant negative correlations were also observed between most PC-to-LPC ratios and age across all participants (Supplementary Table 3). None of the identified circulating metabolites was significantly correlated with BMI (Supplementary Table 4).

### Evaluation of the Metabolite Signature Panel as a Predictor to Categorise Cortical PET-Aβ Status in Cognitively Unimpaired Older Adults

**Fig. 5:**
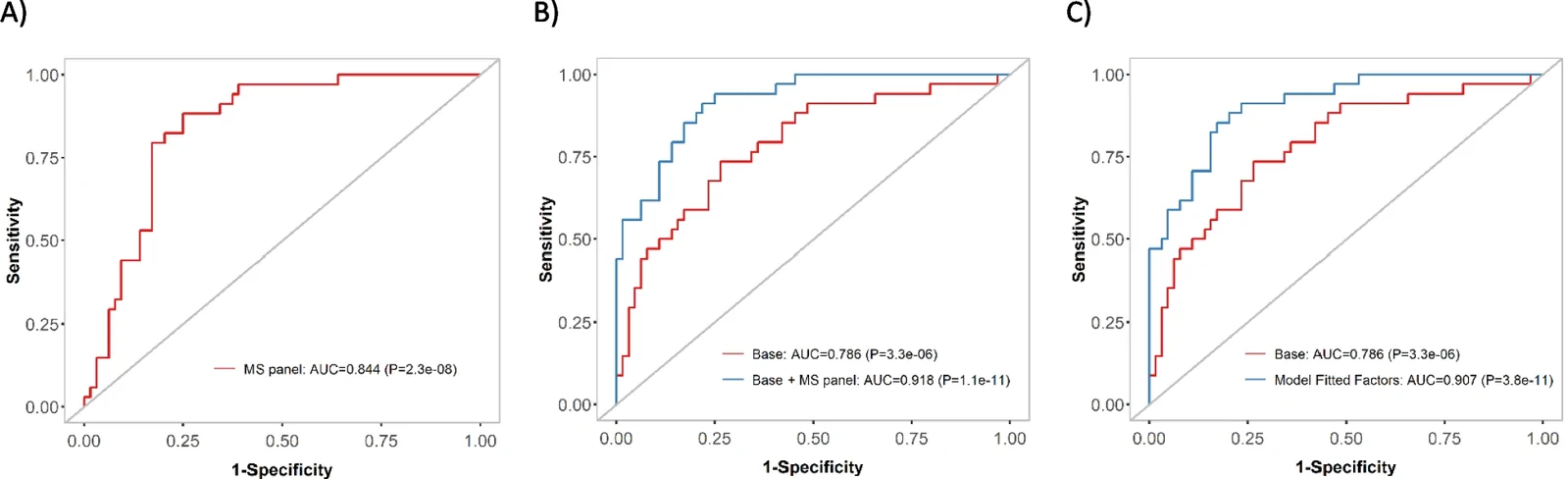
Receiver operating characteristics with cortical Aß status based on FBB-PET (SUVR cut-off: 1.35) in cognitively unimpaired older adults (n= 98) for (A) metabolite signatures, including PC(32:1)/LPC(17:1), PC(32:2)/LPC(17:1), PC(32:3)/LPC(17:1), PC(34:1)/LPC(17:1), PC(34:2)/LPC(17:1), PC(34:3)/LPC(17:1), PC(34:4)/LPC(17:1), PC(34:5)/LPC(17:1), PC(36:1)/LPC(17:1), PC(36:2)/LPC(17:1), PC(36:5)/LPC(17:1), PC(36:6)/LPC(17:1), PC(38:2)/LPC(17:1), PC(40:5)/LPC(17:1), PC(42:5)/LPC(17:1), PC(44:1)/LPC(17:1) and Cer(43:1) (B) Base and Base+metabolite signatures, and (C) Model Fitted factors, incorporating *APOE* ε4 status, sex, age, PC(32:3)/LPC(17:1), PC(34:1)/LPC(17:1), PC(34:2)/LPC(17:1), PC(34:4)/LPC(17:1), PC(34:5)/LPC(17:1), PC(36:1)/LPC(17:1), PC(36:2)/LPC(17:1) and Cer(43:1). Base: *APOE* ε4 status, sex, age, BMI. *AUC* area under the curve, *MS* metabolite signatures

The receiver operating characteristic (ROC) to assess an initial panel of the identified circulating metabolites, including PC(32:1)/LPC(17:1), PC(32:2)/LPC(17:1), PC(32:3)/LPC(17:1), PC(34:1)/LPC(17:1), PC(34:2)/LPC(17:1), PC(34:3)/LPC(17:1), PC(34:4)/LPC(17:1), PC(34:5)/LPC(17:1), PC(36:1)/LPC(17:1), PC(36:2)/LPC(17:1), PC(36:5)/LPC(17:1), PC(36:6)/LPC(17:1), PC(38:2)/LPC(17:1), PC(40:5)/LPC(17:1), PC(42:5)/LPC(17:1), PC(44:1)/LPC(17:1) and Cer(43:1), was investigated to evaluate the classification performance in discriminating cortical PET-Aβ status (Fig. 5). The panel significantly distinguished CU Aβ+ from CU Aβ− individuals with 84.4% AUC (95% CI 77–92%) in cognitively unimpaired older adults (Fig. 5A, Table 4). Further, this metabolite signature panel, in a linear combination with a base model comprising AD-related confounding variables, i.e. *APOE* ε4 status, sex, age and BMI, significantly outperformed in discriminating cortical PET-Aβ status with a higher AUC of 91.8% (95% CI 87–97%, *n* = 98, DeLong’s test *p*-value = 0.0057, *Z* = −2.762) than that predicted by the base model alone (AUC = 78.6%, 95% CI 69–88%) (Fig. 5B, Table 4).

**Table 4:** Receiver operating characteristics analysis parameters at Youden’s index, including AUC with its 95% CI, *p*-value, sensitivity, specificity, threshold, PPV, NPV and accuracy, for each of the biomarker models

	AUC (95% CI)	*p*-value	Sensitivity	Specificity	Threshold	PPV	NPV	Accuracy
Base	78.6% (69–88%)	< 0.00001	73.53	73.44	0.284	59.52	83.93	73.47
MS panel	84.4% (77–92%)	< 0.0000001	88.24	75.00	0.341	65.22	92.31	79.59
Base + MS panel	91.8% (87–97%)	< 0.0000000001	91.18	78.12	0.314	68.89	94.34	82.65
MFF panel	90.7% (85–96%)	< 0.0000000001	85.29	82.81	0.358	72.50	91.38	83.67

Base: *APOE* ε4 status, sex, age, BMI. *MS* metabolite signatures, *MFF* model-fitted factors, *AUC* area under the curve, *CI* confidence interval, *PPV* positive predictive value, *NPV* negative predictive value

Finally, a stepwise logistic regression (stepAIC) procedure was applied to avoid model overfitting while retaining the best predictive factors for forecasting PET-Aβ status. Following this selection procedure, a model comprising *APOE* ε4 status, sex, age, PC(32:3)/LPC(17:1), PC(34:1)/LPC(17:1), PC(34:2)/LPC(17:1), PC(34:4)/LPC(17:1), PC(34:5)/LPC(17:1), PC(36:1)/LPC(17:1), PC(36:2)/LPC(17:1) and Cer(43:1) was chosen. A ROC using the resulting model-fitted values to distinguish CU Aβ+ individuals from CU Aβ− individuals demonstrated better performance (AUC = 90.7%, 95% CI 85–96%, *n* = 98, DeLong’s test *p*-value = 0.0097, *Z* = −2.586) than the base model (Fig. 5C, Table 4) and statistically equivalent to the larger model (*p* > 0.05). Furthermore, to facilitate a comparative evaluation of the biomarker models, all ROC analysis parameters at Youden’s index—including AUC with its 95% CI, *p*-value, sensitivity, specificity, threshold, positive predictive value (PPV), negative predictive value (NPV) and accuracy—are presented in Table 4.

## Discussion

In the current study, we investigated alterations in circulating metabolites associated with early cortical amyloidosis before symptom onset, using a cognitively unimpaired (CU) older adult cohort and targeted metabolite profiling. In this study, significant between-group differences were observed in circulating phosphatidylcholine (PC), lysophosphatidylcholine (LPC) and ceramide (Cer) metabolite classes, wherein, PC(32:1), PC(32:2), PC(32:3), PC(34:1), PC(34:2), PC(34:3), PC(34:4), PC(34:5), PC(36:1), PC(36:2), PC(36:5), PC(36:6), PC(38:2), PC(40:5), PC(42:5) and PC(44:1) levels were reduced, while LPC(17:1) and Cer(43:1) levels were elevated in CU Aβ+ individuals compared to healthy controls (CU Aβ−). These findings indicate alterations in PC, LPC and Cer-mediated biochemical pathways accompanying early cortical amyloidosis, thereby proposing these lipid metabolites as early peripheral fingerprints that mirror preclinical neuropathogenesis. Notably, the current findings align with previous studies that reported depleted plasma PC levels [57, 58] and elevated plasma Cer levels [57, 59] in individuals with AD compared to healthy controls. Further, in a study investigating longitudinal alterations in plasma-based choline phospholipids associated with AD, Dorninger et al. [60] reported markedly elevated levels of LPC in AD participants compared to non-dementia-aged controls. Earlier, in a study on plasma-based lipid metabolites for diagnosing AD, Klavins and Colleagues [61] described lower PC levels and higher LPC levels in individuals with AD and/or MCI compared to controls. Additionally, they observed greater statistical significance when analysing PC-to-LPC ratios. Consistent with Klavins et al. [61], the present study found that calculating PC-to-LPC ratios significantly enhanced the distinction between CU Aβ+ and CU Aβ− individuals, both before and after adjusting for AD-related confounding variables, and following false discovery rate corrections.

Further, upon examination across the spectrum of cortical amyloid deposition, cognitive performance and plasma P-tau181 levels, the identified lipid metabolites showed significant differential associations in CU older adults, even after corrections for false discovery rate**.** While investigating cortical amyloid deposition, PC-to-LPC ratios and Cer(43:1) levels significantly altered across FBB-PET tertiles. Specifically, lower PC-to-LPC ratios and higher Cer(43:1) levels were observed in individuals with higher FBB-PET SUVRs. These findings suggest cross-sectional links between cortical Aβ aggregation and systemic lipid alterations, highlighting their clinical significance to AD pathogenesis. Additionally, while examining cognitive performance, significant positive associations were observed between PC-to-LPC ratios and global composite tertiles. Specifically, lower PC-to-LPC ratios were associated with lower global composite *z*-scores and elevated P-tau181 levels, highlighting their potential relevance to cognitive performance and AD-related pathology. These findings align with Simpson et al. [62], who reported an association between reduced PC levels and poor memory function in healthy older adults. Furthermore, lower PC-to-LPC ratios were associated with elevated plasma P-tau181 levels, suggesting their potential relevance to plasma P-tau181 elevation in CU older adults, representing a novel finding. As plasma P-tau181 is closely linked to cerebral amyloid pathology, the observed metabolite–P-tau181 associations may partly reflect underlying amyloid burden. Given the preclinical, exploratory nature of this study and modest sample size, adjustment for amyloid measures was not performed. Future studies in larger, well-characterised cohorts spanning the AD continuum should determine whether these associations remain independent after accounting for cerebral amyloid burden.

Moreover, when assessing the receiver operating characteristics (ROC) for the potential to classify cortical PET-Aβ status in CU older adults, the base model showed moderate diagnostic performance (AUC = 78.6%, 95% CI 69–88%), while the metabolite signature panel alone achieved a higher AUC of 84.4% (95% CI 77–92%) with improved sensitivity (88.24%) and specificity (75%). Combining the metabolite signatures with the base yielded the highest diagnostic performance, achieving an AUC of 91.8% (95% CI, 87–97%), indicating excellent classification accuracy. This combined model also achieved excellent sensitivity (91.18%) and NPV (94.34%). Finally, this study proposed a refined model incorporating fitted factors, including *APOE* ε4 status, sex, age, PC(32:3)/LPC(17:1), PC(34:1)/LPC(17:1), PC(34:2)/LPC(17:1), PC(34:4)/LPC(17:1), PC(34:5)/LPC(17:1), PC(36:1)/LPC(17:1), PC(36:2)/LPC(17:1) and Cer(43:1), which demonstrated robust diagnostic performance with an AUC of 90.7% (95% CI 85–96%), and a well-balanced sensitivity of 85.29% and specificity of 82.81% at the optimal threshold of 0.358. Collectively, these findings underscore the utility of these metabolite signatures in identifying CU individuals with early amyloid positivity. Interestingly, Mapstone et al. [63] identified a panel of ten plasma metabolites, including acylcarnitines (ACs), PCs and LPCs, that can predict the onset of cognitive impairment in older adults before clinical symptoms emerge.

**Fig. 6:**
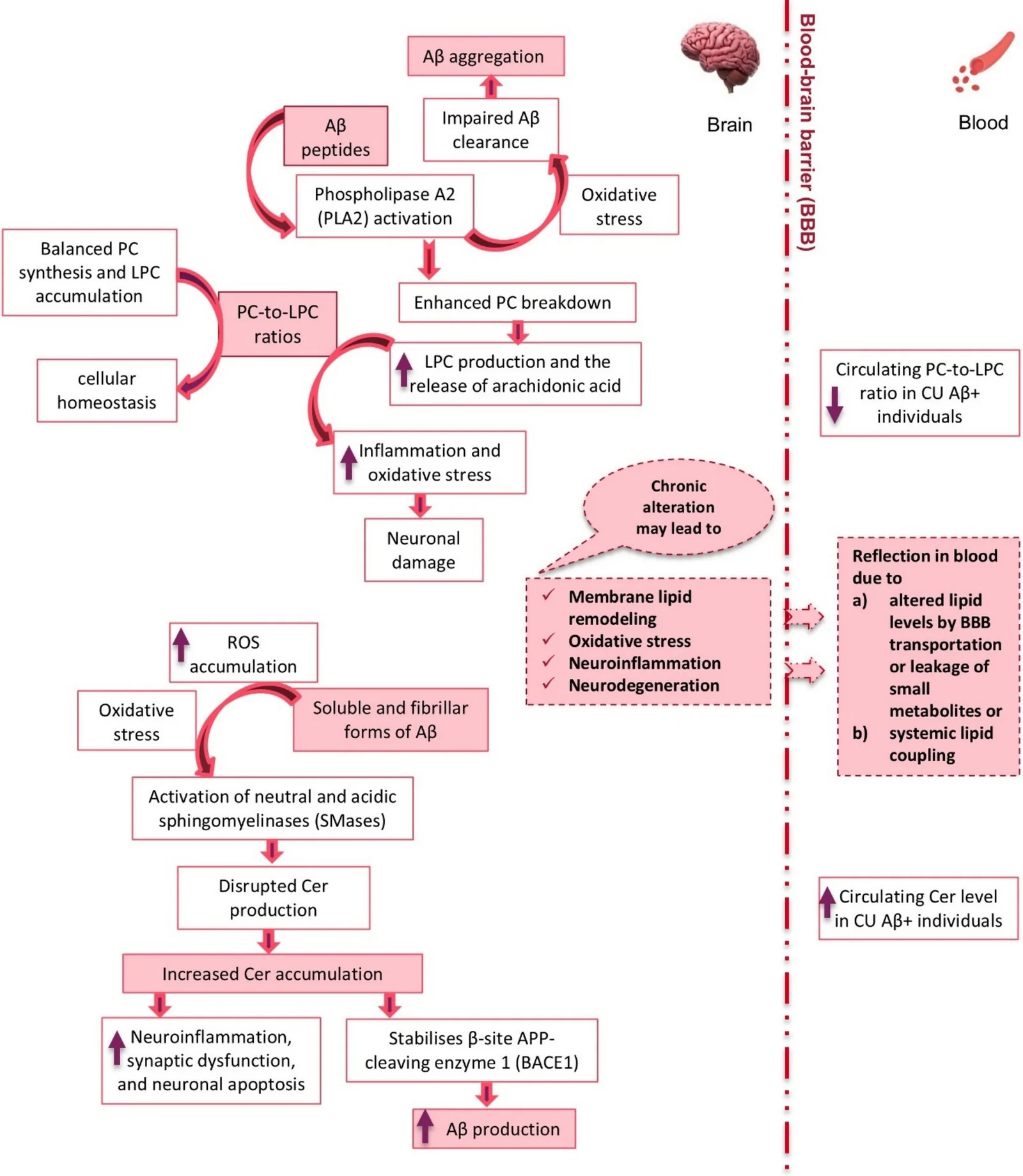
Proposed mechanisms underlying altered PC-to-LPC ratios and Cer levels, and their implications in CU older adults. Aβ production and aggregation may lead to enzyme activation (e.g., PLA2, SMase), resulting in increased PC hydrolysis and LPC production as well as Cer accumulation, thereby exacerbating neuronal damage through enhanced oxidative stress, neuroinflammation, synaptic dysfunction, and neuronal apoptosis, ultimately driving chronic membrane lipid remodeling. Such central lipid perturbations may be reflected in the blood via BBB transport/leakage of small lipid metabolites or systemic lipid coupling, leading to altered plasma PC-to-LPC ratios and Cer levels. *PC* phosphatidylcholine, *LPC* lysophosphatidylcholine, *Cer* ceramide, *ROS* reactive oxygen species

PC and LPC are important phospholipids that play crucial roles in cell membrane structure, lipid metabolism and signal transduction [64–66]. PC, the predominant phospholipid in cell membranes and plasma, serves as a precursor for several key bioactive molecules: (a) LPC, produced through the enzymatic action of phospholipase A2 (PLA2) or lecithin-cholesterol-acyltransferase (LCAT) in the bloodstream, as well as triglyceride lipases in endothelial cells or reactive oxygen species (ROS) and PLA2 in granulocytes, (b) acetylcholine, a neurotransmitter essential for cognitive function and (c) second messengers, including diacylglycerol and phosphatidic acid, which regulate intracellular signalling pathways [64, 66–68]. Nevertheless, previous studies have reported Aβ peptides induced direct activation of PLA2 [69, 70], leading to increased LPC production and the release of arachidonic acid. This process fuels the inflammatory cascade, ultimately exacerbating AD pathogenesis [71–73]. Besides, elevated PLA2 activity may further facilitate Aβ aggregation and toxicity through oxidative stress–induced impaired Aβ clearance, as described by Somin et al. [74]. Additionally, LPC can be converted back to PC through reacylation by LPC-acyltransferase (LPCAT); thus, a reduction in reacylation may lead to elevated LPC levels. At sites of inflammation, LPC regulates the immune response by attracting T cells, monocytes and neutrophils, while also activating inflammation-related pathways [67, 75]. Accordingly, the PC-to-LPC ratio reflects the balance between PC synthesis and LPC accumulation, which is essential for maintaining cellular homeostasis. A reduction in PC synthesis and increased degradation by phospholipases can lead to LPC accumulation, while elevated LPC levels may promote inflammation and oxidative stress, further exacerbating neuronal damage. Therefore, the decline in circulating PC-to-LPC ratios in CU Aβ+ individuals likely reflects enhanced PC breakdown and inflammation, potentially representing peripheral metabolic responses to central amyloid pathology (Fig. 6).

Cers are the central bioactive molecules of sphingolipid biosynthesis and catabolism pathways [76, 77]. Sphingolipids are structural components of dynamic membrane microdomains and play active roles in numerous signalling pathways as second messengers [78, 79]. These sphingolipids, at their normal levels, help inhibit the production and aggregation of Aβ peptides, thus impeding AD progression [73, 80]. Cer synthesis involves either catabolic degradation of sphingomyelin (SM) by the action of the sphingomyelinase (SMase) enzyme or the de novo synthesis pathway in the endoplasmic reticulum through condensation of serine and palmitoyl-CoA by the action of serine palmitoyltransferase enzyme or the salvage pathway, wherein sphingosine, a product of sphingolipid catabolism, undergoes reacylation to produce Cer [76, 77, 81]. Further, numerous studies have consistently reported higher Cer levels in the brains and CSF of AD individuals [82–85]; additionally, Cutler et al. [83] illustrated disrupted Cer production as a consequence of Aβ-induced oxidative stress in the membrane. In addition, Lee and colleagues [84] demonstrated that Aβ induces Cer-dependent oligodendrocyte death, which is associated with the activation of neutral SMase and elevated Cer levels. Further, in a high-fat diet setting, Patil et al. [86] detected elevated astroglial Cer production through de novo synthesis, leading to higher Aβ peptide synthesis and tau hyperphosphorylation. Also, Katsel et al. [87] reported that genes associated with the de novo synthesis of sphingolipids exhibit elevated expression early in AD progression. Furthermore, research has shown that Cers elevate Aβ levels by enhancing amyloid precursor protein (APP) processing by stabilising β-site APP-cleaving enzyme 1 (BACE1); additionally, both soluble and fibrillar forms of Aβ can trigger the activation of neutral and acidic SMases by increasing ROS accumulation, leading to a cycle of Cer accumulation and Aβ production [73, 76, 88–90]. Besides, evidence supports that plasma Cers are reflective of disturbances in sphingolipid metabolism occurring in both the periphery and brain during AD. For example, studies have shown that AD individuals exhibit elevated levels of specific Cer species in their plasma compared to healthy controls [19, 57], and the elevated Cer levels were linked to cognitive decline and disease progression. Moreover, dysregulated sphingolipid metabolism, as reflected in increased plasma Cers, contributes to neuroinflammation, synaptic dysfunction and neuronal apoptosis [88]. Taken together with previous evidence, increased circulating Cers may indicate mechanisms underlying or resulting from toxic Aβ formation and accumulation, while these peripheral alterations may also represent systemic metabolic reflections/adaptations to central neuropathological processes (Fig. 6).

The current investigation on circulating metabolite alterations for preclinical AD has several strengths and weaknesses. The strengths of this study include the precise quantification of plasma metabolites using the Biocrates AbsoluteIDQ® p400 HR platform and the comprehensive evaluation of metabolite signatures in relation to AD-related markers, including amyloid-PET imaging, cognitive performance and plasma P-tau181 levels. Another strength is the use of a structured, biologically informed analytical framework that combines exploratory metabolite screening, biologically relevant metabolite ratio analyses and adjusted confirmatory modelling with false discovery rate correction, enabling both hypothesis generation and robust evaluation of the findings. The study also included comprehensive ROC analyses, assessment of diagnostic performance metrics and comparison of biomarker models using DeLong’s test. Collectively, these approaches enhanced the reliability, biological relevance and potential clinical utility of the identified metabolite signatures associated with early AD pathology. Despite its strengths, the current study has certain inherent limitations, including a modest sample size, a cross-sectional design that focuses solely on presymptomatic participants, a deficit in considering CSF metabolite levels, challenges in adjusting for differences in race and ethnicity and the inability to account for the effects of diet, medications and lifestyle factors. Furthermore, the findings require validation in independent cohorts to confirm the robustness and generalisability of the proposed biomarker models.

In conclusion, the current study is the first to demonstrate reduced circulating PC-to-LPC ratios and elevated Cer(43:1) levels in CU individuals with early cortical amyloidosis ahead of symptom manifestations. These alterations highlight systemic lipid dysregulation consistent with membrane destabilisation, synaptic dysfunction, oxidative stress and neuroinflammatory cascades—hallmark mechanisms of AD neuropathogenesis. Importantly, lower PC-to-LPC ratios were positively associated with poorer cognitive composite scores, suggesting their potential relevance for identifying individuals with weaker cognitive performance. Furthermore, the association between lower PC-to-LPC ratios and elevated plasma P-tau181 levels highlights their potential as biomarkers associated with plasma P-tau181 elevation in CU older adults. Moreover, metabolite-based panels incorporating these circulating lipid metabolites significantly distinguished cortical PET-Aβ status in CU older adults with excellent discriminative performance (AUC > 90%) and high reliability in ruling out amyloid negativity (NPV > 90%). Taken together, these metabolite signatures represent promising non-invasive biomarkers associated with early amyloid pathology and may provide mechanistic insights into the biochemical cascade underlying preclinical AD. However, further cross-sectional and longitudinal investigations using the same analytical platform and larger independent cohorts, incorporating parallel CSF and plasma metabolite measurements and robust validation approaches, such as cross-validation or bootstrap resampling, are needed to validate the current observations.

## Supplementary Information

Below is the link to the electronic supplementary material.

ESM 1 (PDF 205 KB)

## Data Availability

The dataset used and analysed in this study is available upon reasonable request from the corresponding author. However, it is not publicly accessible due to privacy and ethical restrictions.
