# Supplementary material for: Circulating Metabolite Signatures Associated With Amyloid Positivity, Cognitive Performance and Plasma P-tau181 Levels in Cognitively Unimpaired Older Adults
Source: Mol Neurobiol. 2026 Sep 14;63(1):892. doi: 10.1007/s12035-026-06184-1 (PMC13572340; doi:10.1007/s12035-026-06184-1)
Supplement: Supplementary file 1 [file 12035_2026_6184_MOESM1_ESM.pdf]

Circulating metabolite signatures associated with amyloid positivity, cognitive performance and plasma P-tau181 levels in cognitively unimpaired older adults

Tahmida Sharmin [1, 2], James D. Doecke [3, 4], Pratishtha Chatterjee [1, 5, 6], Hamid R. Sohrabi [1, 7, 8, 9], Henrik Zetterberg[10, 11, 12, 13, 14, 15, 16, 17], Nicholas J. Ashton[10, 18], Manohar L. Garg[1, 19], Kaj Blennow[10, 11], Ralph N. Martins[1, 4, 7, 9, 19]

[1] Macquarie Medical School, Macquarie University, Macquarie Park, New South Wales, Australia
[2] Department of Pharmacy, University of Rajshahi, Rajshahi, Bangladesh
[3] Australian eHealth Research Centre, CSIRO, Brisbane, Queensland, Australia
[4] School of Medical and Health Sciences, Edith Cowan University, Perth, Western Australia, Australia
[5] Florey Institute of Neuroscience and Mental Health, University of Melbourne, Victoria, Australia
[6] Department of Neuroscience, Monash University, Victoria, Australia
[7] Centre for Healthy Ageing, Health Futures Institute, Murdoch University, Murdoch, Western Australia, Australia
[8] School of Psychology, Murdoch University, Murdoch, Western Australia, Australia
[9] Alzheimer's Research Australia, Nedlands, Western Australia, Australia
[10] Department of Psychiatry and Neurochemistry, University of Gothenburg, Mölndal, Sweden
[11] Clinical Neurochemistry Laboratory, Sahlgrenska University Hospital, Mölndal, Sweden
[12] Department of Neurodegenerative Disease, Institute of Neurology, UCL, Queen Square, London, UK
[13] UK Dementia Research Institute, UCL, London, UK
[14] Hong Kong Center for Neurodegenerative Diseases, Clear Water Bay, Hong Kong, China
[15] Wisconsin Alzheimer's Disease Research Center, University of Wisconsin School of Medicine and Public Health, University of Wisconsin-Madison, Madison, Wisconsin, USA
[16] Department of Pathology and Laboratory Medicine, University of Wisconsin School of Medicine and Public Health, University of Wisconsin-Madison, Madison, Wisconsin, USA
[17] Centre for Brain Research, Indian Institute of Science, Bangalore, India
[18] Department of Old Age Psychiatry, Institute of Psychiatry, Psychology & Neuroscience, King's College London, London, UK
[19] Lifespan Health and Wellbeing Research Centre, Macquarie University, Macquarie Park, New South Wales, Australia

Correspondence: Professor Ralph N. Martins, Macquarie Medical School, Macquarie University, Macquarie Park, NSW 2109, Australia. Email: ralph.martins@mq.edu.au, Work phone: +61-2-9850-4573.

Supplementary Table 1 Comparison of circulating metabolite signatures between *APOE* ε4- and *APOE* ε4+ individuals

Plasma metabolites (μM)	N	*APOE* ε4-	*APOE* ε4+	p-value
PC(32:1)/LPC(17:1)	79, 21	130.376 ± 64.972	125.534 ± 99.268	0.288
PC(32:2)/LPC(17:1)	79, 21	26.242 ± 12.865	21.389 ± 10.705	0.122
PC(32:3)/LPC(17:1)	79, 21	6.451 ± 2.921	5.865 ± 2.729	0.490
PC(34:1)/LPC(17:1)	79, 21	1452.754 ± 547.148	1213.244 ± 504.087	**0.035**
PC(34:2)/LPC(17:1)	79, 21	2151.076 ± 847.347	1709.681 ± 660.629	**0.014**
PC(34:3)/LPC(17:1)	79, 21	96.338 ± 38.109	84.771 ± 42.634	0.161
PC(34:4)/LPC(17:1)	79, 21	33.837 ± 15.132	37.061 ± 33.165	0.790
PC(34:5)/LPC(17:1)	79, 21	9.123 ± 4.864	7.893 ± 4.747	0.269
PC(36:1)/LPC(17:1)	79, 21	236.498 ± 89.324	213.566 ± 87.610	0.213
PC(36:2)/LPC(17:1)	79, 21	1019.441 ± 426.555	829.764 ± 340.884	**0.027**
PC(36:5)/LPC(17:1)	79, 21	685.050 ± 306.678	596.269 ± 295.321	0.154
PC(36:6)/LPC(17:1)	79, 21	24.592 ± 11.148	22.103 ± 13.519	0.194
PC(38:2)/LPC(17:1)	79, 21	21.226 ± 11.791	17.902 ± 11.889	0.200
PC(40:5)/LPC(17:1)	79, 21	62.462 ± 23.551	54.830 ± 19.011	0.166
PC(42:5)/LPC(17:1)	79, 21	3.433 ± 1.721	2.809 ± 2.446	**0.020**
PC(44:1)/LPC(17:1)	79, 19	27.311 ± 20.327	24.821 ± 19.964	0.453
Cer(43:1) $	79, 21	0.469 ± 0.193	0.499 ± 0.223	0.744

Comparisons between *APOE* ε4- and *APOE* ε4+ individuals were performed using the nonparametric Mann–Whitney U test or independent-samples t-test, as appropriate. '$' represents the logarithmic transformation to meet the requirement for normal distribution and p-values < 0.05 were considered statistically significant and are shown in **bold**.

Supplementary Table 2 Comparison of circulating metabolite signatures between male and female participants

Plasma metabolites (μM)	N	Male	Female	p-value
PC(32:1)/LPC(17:1)	32, 68	100.624 ± 46.171	142.881 ± 79.344	**0.001**
PC(32:2)/LPC(17:1)	32, 68	19.151 ± 10.531	28.081 ± 12.470	**0.000105**
PC(32:3)/LPC(17:1)	32, 67	4.393 ± 1.804	7.238 ± 2.847	**0.000001**
PC(34:1)/LPC(17:1)	32, 68	1276.382 ± 561.937	1461.786 ± 530.387	0.058
PC(34:2)/LPC(17:1)	32, 68	1788.801 ± 803.806	2185.245 ± 815.113	**0.014**
PC(34:3)/LPC(17:1)	32, 68	73.450 ± 25.416	103.536 ± 40.909	**0.000035**
PC(34:4)/LPC(17:1)	32, 68	24.676 ± 8.919	39.143 ± 22.197	**0.000029**
PC(34:5)/LPC(17:1)	32, 68	6.124 ± 2.726	10.154 ± 5.093	**0.000018**
PC(36:1)/LPC(17:1)	32, 68	190.373 ± 74.483	251.122 ± 89.133	**0.001**
PC(36:2)/LPC(17:1)	32, 68	801.347 ± 370.503	1063.496 ± 411.805	**0.001**
PC(36:5)/LPC(17:1)	32, 68	507.627 ± 193.210	741.126 ± 320.155	**0.000348**
PC(36:6)/LPC(17:1)	32, 68	17.028 ± 6.330	27.382 ± 12.142	**0.000017**
PC(38:2)/LPC(17:1)	32, 68	13.869 ± 7.884	23.661 ± 12.120	**0.000137**
PC(40:5)/LPC(17:1)	32, 68	47.943 ± 13.557	66.937 ± 23.787	**0.000017**
PC(42:5)/LPC(17:1)	32, 68	2.953 ± 1.696	3.466 ± 1.978	0.165
PC(44:1)/LPC(17:1)	31, 67	3.274 ± 1.939	4.271 ± 2.194	0.390
Cer(43:1) $	32, 68	0.482 ± 0.236	0.472 ± 0.182	0.929

Comparisons between male and female participants were performed using the nonparametric Mann–Whitney U test or independent-samples t-test, as appropriate. '$' represents the logarithmic transformation to meet the requirement for normal distribution and p-values < 0.05 were considered statistically significant and are shown in **bold**.

Supplementary Table 3 Correlations between circulating metabolite signatures and age in all participants

Plasma metabolites (µM)	Age	
	ρ	p-value
PC(32:1)/LPC(17:1)	-0.088	0.386
PC(32:2)/LPC(17:1)	-0.359	**0.000241**
PC(32:3)/LPC(17:1)	-0.236	**0.018**
PC(34:1)/LPC(17:1)	-0.139	0.168
PC(34:2)/LPC(17:1)	-0.304	**0.002**
PC(34:3)/LPC(17:1)	-0.268	**0.007**
PC(34:4)/LPC(17:1)	-0.239	**0.017**
PC(34:5)/LPC(17:1)	-0.308	**0.002**
PC(36:1)/LPC(17:1)	-0.296	**0.003**
PC(36:2)/LPC(17:1)	-0.355	**0.000287**
PC(36:5)/LPC(17:1)	-0.337	**0.001**
PC(36:6)/LPC(17:1)	-0.290	**0.003**
PC(38:2)/LPC(17:1)	-0.283	**0.004**
PC(40:5)/LPC(17:1)	-0.234	**0.019**
PC(42:5)/LPC(17:1)	-0.090	0.371
PC(44:1)/LPC(17:1)	-0.177	0.082
Cer(43:1) $	0.060	0.551

The correlations between the circulating metabolite signatures and age were calculated using Spearman correlation. 'ρ' represents the Spearman correlation coefficient, and p-values < 0.05 were considered statistically significant and are shown in **bold**. '$' represents the logarithmic transformation to meet the requirement for normal distribution. For PC(44:1)/LPC(17:1), n = 98; for all other metabolites, n = 100.

Supplementary Table 4 Correlations between circulating metabolite signatures and body mass index (BMI) in all participants

Plasma metabolites (µM)	BMI	
	ρ	p-value
PC(32:1)/LPC(17:1)	-0.007	0.946
PC(32:2)/LPC(17:1)	-0.104	0.302
PC(32:3)/LPC(17:1)	-0.094	0.351
PC(34:1)/LPC(17:1)	-0.049	0.626
PC(34:2)/LPC(17:1)	-0.162	0.107
PC(34:3)/LPC(17:1)	-0.077	0.447
PC(34:4)/LPC(17:1)	-0.042	0.677
PC(34:5)/LPC(17:1)	-0.121	0.230
PC(36:1)/LPC(17:1)	-0.084	0.407
PC(36:2)/LPC(17:1)	-0.117	0.246
PC(36:5)/LPC(17:1)	-0.123	0.222
PC(36:6)/LPC(17:1)	-0.055	0.586
PC(38:2)/LPC(17:1)	-0.077	0.444
PC(40:5)/LPC(17:1)	-0.048	0.635
PC(42:5)/LPC(17:1)	-0.112	0.267
PC(44:1)/LPC(17:1)	0.144	0.156
Cer(43:1) $	0.067	0.508

The correlations between the circulating metabolite signatures and BMI were calculated using Spearman correlation. 'ρ' represents the Spearman correlation coefficient, and p-values < 0.05 were considered significant. '$' represents the logarithmic transformation to meet the requirement for normal distribution. For PC(44:1)/LPC(17:1), n = 98; for all other metabolites, n = 100.
